# Optimizing Revascularization in Ischemic Cardiomyopathy: Comparative Evidence on the Benefits and Indications of CABG and PCI

**DOI:** 10.3390/life15040575

**Published:** 2025-04-01

**Authors:** Dan M. Prunea, Calin Homorodean, Maria Olinic, Alexandru Achim, Dan-Mircea Olinic

**Affiliations:** 1 Medical Clinic No. 1, “Iuliu Hatieganu” University of Medicine and Pharmacy, 400006 Cluj-Napoca, Romania; 2 Cluj County Emergency Clinical Hospital, 3-5, Clinicilor Street, 400006 Cluj-Napoca, Romania; 3 “Niculae Stăncioiu” Heart Institute, “Iuliu Hatieganu” University of Medicine and Pharmacy, 400001 Cluj-Napoca, Romania

**Keywords:** ischemic cardiomyopathy, left ventricular dysfunction, heart failure, myocardial revascularization, percutaneous coronary intervention, coronary artery bypass grafting, myocardial viability, REVIVED, STICH

## Abstract

Ischemic cardiomyopathy remains a leading cause of heart failure, yet the optimal revascularization approach for patients with reduced left ventricular function remains uncertain. This review synthesizes current evidence on coronary revascularization strategies, emphasizing real-world applicability and individualized treatment. It critically evaluates the benefits and limitations of coronary artery bypass grafting [CABG] and percutaneous coronary intervention [PCI], highlighting key knowledge gaps. Findings from the STICH trial demonstrate that CABG improves long-term survival despite an elevated early procedural risk, particularly in patients with extensive multivessel disease. In contrast, the REVIVED-BCIS2 trial suggests that PCI enhances quality of life but does not significantly reduce mortality compared to optimal medical therapy, making it a viable alternative for high-risk patients ineligible for surgery. This review underscores the role of advanced imaging techniques in myocardial viability assessment and emphasizes the importance of comprehensive risk stratification in guiding revascularization decisions. Special attention is given to managing high-risk patients unsuitable for CABG and the potential benefits of PCI in symptom relief despite uncertain survival benefits. A stepwise algorithm is proposed to assist clinicians in tailoring revascularization strategies, reinforcing the need for a multidisciplinary Heart Team approach to optimize outcomes.

## 1. Introduction

Ischemic cardiomyopathy (ICM) due to coronary artery disease (CAD) is the primary underlying cause of heart failure (HF) in wealthy nations, particularly among aging populations with a higher burden of comorbidities [1]. The term describes left ventricular dysfunction (LVD) resulting from significant CAD; clinical trials and recent registries have attributed worse outcomes to ICM compared with non-ischemic etiologies [2,3]. The management of patients with CAD and reduced left ventricular ejection fraction (LVEF) poses a significant clinical and public health challenge [4].

Therapeutic options for ICM involve a comprehensive approach that begins with lifestyle modifications and medical therapy as the cornerstones of treatment and extends to adjuncts such as coronary revascularization, mechanical circulatory support (MCS), or cardiac transplantation [5,6].

In the context of a presumed causal relationship between CAD and the onset of ischemic LVD, there has traditionally been a reliance on coronary revascularization to restore myocardial blood flow, improve symptoms, and potentially reverse LVD, even though evidence supporting the reversibility of myocardial hibernation remains limited [7].

In patients with severely reduced LVEF, percutaneous coronary intervention (PCI) represents a less invasive revascularization option, especially for those at high surgical risk, and its use is increasing in clinical practice. Despite its appeal, the benefits of PCI over optimal medical therapy (OMT) remain uncertain. Studies have reported that although PCI is the preferred strategy in 40–50% of ICM cases, it may be associated with higher mortality and increased rates of major adverse cardiovascular events (MACE) compared with coronary artery bypass grafting (CABG) [8,9].

Patients with low to intermediate surgical risk and complex CAD are deemed suitable for CABG regardless of LVEF, according to current guidelines [10,11,12]. However, many patients, due to advanced age, significant comorbidities, frailty, or unfavorable coronary anatomy, are not candidates for CABG. For these individuals, alternative strategies such as PCI combined with OMT become particularly relevant [10,13].

**Figure 1:**
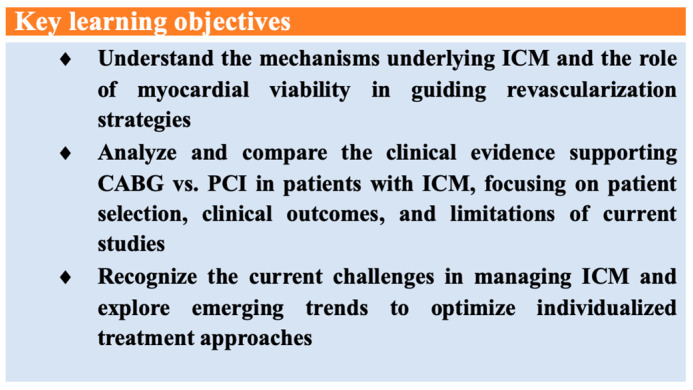
Key learning objectives. CABG, coronary artery bypass grafting; ICM, ischemic cardiomyopathy; PCI, percutaneous coronary intervention.

This study aims to integrate current evidence on revascularization in ICM, focusing on real-world applicability, to determine the optimal management strategy by comparing CABG and PCI, thereby guiding individualized treatment decisions for all patient groups, including those ineligible for CABG (Figure 1).

## 2. Pathophysiology and Rationale for Revascularization

### 2.1. Mechanisms of Ischemic Left Ventricular Dysfunction

**Figure 2:**
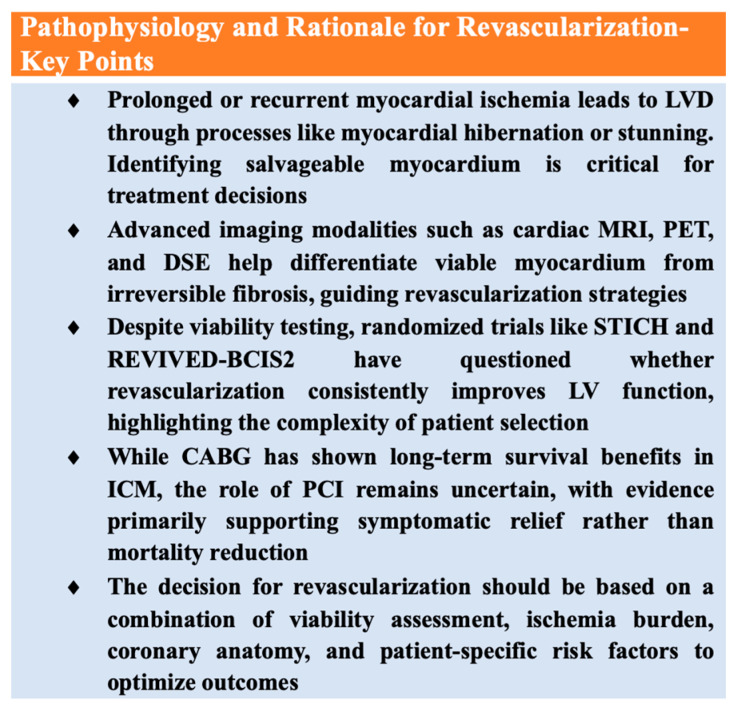
Pathophysiology and rationale for revascularization in ischemic cardiomyopathy. DSE, dobutamin stress echocardiography; ICM, ischemic cardiomyopathy; LV, left ventricular; LVD, left ventricular dysfunction; MRI, magnetic resonance imaging; PCI, percutaneous coronary intervention; PET, positron emission tomography; REVIVED-BCIS2, Study of Efficacy and Safety of Percutaneous Coronary Intervention to Improve Survival in Heart Failure; STICH, Surgical Treatment for Ischemic Heart Failure.

Under physiological conditions, myocardial contraction is powered by oxygen delivered through adequate perfusion [14]. As CAD develops, prolonged or recurrent episodes of myocardial ischemia can lead to myocardial hibernation, stunning, or even acute myocardial infarction (MI), ultimately resulting in impaired contractility and, in advanced cases, severe LVD [15] (Figure 2).

Hibernation is an adaptive response to chronic ischemia in which cell survival is prioritized over contractile function. It typically occurs in advanced CAD, where the extent of the disease is sufficient for ischemia to occur even during routine daily activities [16]. Recent evidence suggests that interventions aimed to improve coronary perfusion can potentially reverse myocardial hibernation, challenging earlier beliefs about an unequivocal link between revascularization and hibernation [17].

Another reversible state of myocardial dysfunction is stunned myocardium, which differs from hibernation in that it does not require any intervention to regain function. In stunned myocardium, contractile function recovers gradually despite restored coronary perfusion [18]. Even short episodes of ischemia can impair left ventricular (LV) function for several hours without causing irreversible damage; this phenomenon is observed in both acute and chronic settings [19]. Finally, MI represents a major and often irreversible cause of ICM due to the loss of viable tissue [20].

ICM thus involves a complex interplay of physiological, molecular, and structural changes that lead to LV remodeling. Early remodeling is characterized by wall thinning and dilation, whereas later stages result in fibrosis and scarring [21]. Consequently, understanding and differentiating salvageable myocardium from tissue that has undergone irreversible changes is crucial for selecting the optimal revascularization strategy.

### 2.2. Myocardial Viability and Its Role in Decision-Making

Myocardial viability is one of the essential factors guiding the decision-making process for revascularization. It is defined as the presence of myocardial cells with preserved metabolic activity and intact cell membranes, which in turn indicates a maintained contractile reserve. In other words, viability denotes dysfunctional myocardial segments that retain the potential to recover function following revascularization [22]. Several noninvasive imaging modalities have been developed to assess myocardial viability. Echocardiography, the most frequently used method for determining baseline contractile function, is often complemented by cardiac magnetic resonance imaging (cMRI), which can identify the localization and extent of myocardial scarring. Other metabolic techniques, such as positron emission tomography (PET) and single photon emission computed tomography (SPECT), evaluate cell membrane integrity [23]. Moreover, these modalities are frequently combined with dobutamine stress imaging to identify the presence of contractile reserve [24].

Echocardiography—including three-dimensional (3D) echocardiography, dobutamine stress echocardiography (DSE), and contrast echocardiography—is essential not only for evaluating myocardial viability but also for assessing LV function and thereby diagnosing ICM [25]. For example, a wall thickness of less than 6 mm has been associated with myocardial scarring and a low probability of contractile recovery after revascularization [26].

**Figure 3:**
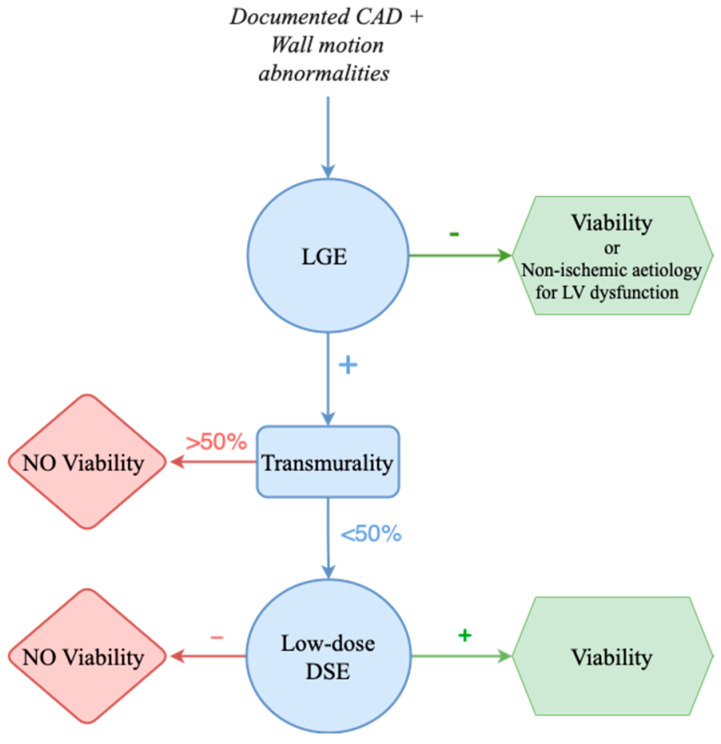
Proposed algorithm for assessing myocardial viability with cMRI. CAD, coronary artery disease; cMRI, cardiac magnetic resonance imaging; DSE, dobutamine stress echocardiography; LGE, late gadolinium enhancement; LV, left ventricle.

Cardiac MRI combines techniques such as late gadolinium enhancement (LGE) and low-dose dobutamine stress MRI to evaluate both myocardial viability and contractile reserve. LGE quantifies nonviable tissue (e.g., scar tissue), whereas low-dose dobutamine stress MRI assesses the potential for functional recovery [27,28]. In patients with more than 50% transmural scarring, the likelihood of recovery diminishes significantly (Figure 3); however, further large-scale trials are needed to clarify the role of cardiac MRI in predicting revascularization outcomes [22].

Finally, SPECT and PET are nuclear imaging techniques used to assess myocardial perfusion and metabolism, thereby differentiating viable from nonviable myocardium. Although SPECT is widely available, its resolution is lower than that of PET, which offers superior dual assessment using both perfusion and metabolic tracers. PET’s ability to detect a perfusion-metabolic mismatch is valuable for identifying hibernating myocardium, although its high cost limits routine use [29].

Despite some limitations, these methods are used for viability testing in clinical practice, not only helping in quantifying the degree of myocardial injury but also informing clinicians on the potential for functional recovery, thereby guiding the selection of patients for either CABG or PCI [22]. Nevertheless, recent randomized trials, such as the REVIVED-BCIS2 (Revascularization for Ischemic Ventricular Dysfunction) trial, have not demonstrated a definitive link between percutaneous revascularization and myocardial viability, calling into question the predictive value of viability for long-term survival and improved clinical outcomes [3]. This dissociation between viability and improved long-term survival underscores that while viability testing remains valuable for assessing myocardial injury and potentially guiding symptomatic management, its role as a stand-alone prognostic tool for mortality reduction, especially in the context of PCI, appears to be more limited than previously assumed. Consequently, while viability assessment remains valuable for establishing the extent of myocardial injury and may aid in guiding therapeutic approaches for symptom relief and functional improvement, its role as a prognostic tool for mortality reduction in the context of PCI should be reconsidered [27].

While registries suggest that viability is associated with improved LV function following revascularization, trials like STICH (Surgical Treatment of Ischemic Heart Failure) and COURAGE have shown no mortality benefit. Furthermore, the STICH viability substudy, which evaluated the impact of viability testing on clinical outcomes in ICM patients undergoing CABG, provided pivotal insights into this issue. Contrary to prior observational data suggesting that viability might predict mortality benefit, the STICH substudy failed to demonstrate a significant interaction between viability status and survival outcomes following revascularization. Patients with viable myocardium did not experience a statistically significant reduction in all-cause mortality compared to those without demonstrable viability, challenging the long-held assumption that viability alone should guide revascularization decisions [30]. It is also important to note that viability predicts responses to various treatments (e.g., pharmacotherapy and cardiac resynchronization therapy), making it difficult to isolate the effect of coronary revascularization [31]. Additionally, randomized trials on chronic total occlusion (CTO) PCI have shown no regional functional improvement even in viable myocardium, although these studies did not focus on patients with severe LV dysfunction [32].

Consequently, taken together, the cumulative evidence from STICH and REVIVED-BCIS2 underscores that viability assessment, while useful for characterizing myocardial injury and guiding symptomatic management, should not be the sole determinant in selecting patients for revascularization. Instead, viability testing should be incorporated into a broader risk stratification framework that considers anatomical complexity, symptom burden, comorbidities, and surgical candidacy [26,33,34].

### 2.3. Potential Benefits of Revascularization

Despite the lack of unequivocal evidence demonstrating that coronary revascularization reverses myocardial hibernation, revascularization has traditionally been considered a viable option for patients with LV dysfunction due to its causal link with CAD. Much of the supporting evidence comes from various observational studies [35,36]. In contrast, contradictory findings also exist: for instance, analyses from the CASS registry have shown benefits in patients with severe CAD and angina [37], while a meta-analysis of 24 studies reported an 80% reduction in mortality for patients with LV dysfunction and viable myocardium undergoing revascularization [7]. Additionally, the PARR-2 trial, which enrolled 430 patients with ICM and LVEF below 35%, demonstrated a significant mortality reduction with PET-guided revascularization [38]. On the other hand, the HEART trial, terminated early due to slow recruitment, which aimed to enroll patients with ischemic cardiomyopathy, LVEF < 35%, and significant myocardial viability, showed no potential inferiority for a conservative management when compared to an invasive approach [39]. Characteristics of the studies discussed in this paper are summarized in Table 1.

Because many of these studies were not designed as dedicated trials for coronary revascularization, their results must be interpreted cautiously due to issues such as non-standardized definitions of viability and selection bias. Their relevance is further questioned by the continuous improvements in medical and device therapies and by evidence from recent randomized trials.

## 3. Evidence for Revascularization in Ischemic Cardiomyopathy: CABG and PCI

**Figure 4:**
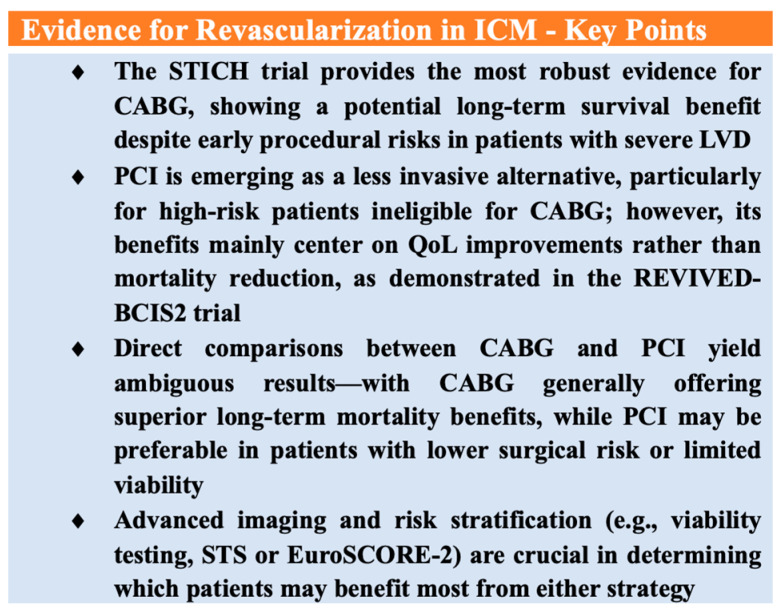
Evidence for revascularization in ischemic cardiomyopathy. CABG, coronary artery bypass grafting; EuroSCORE, European System for Cardiac Operative Risk Evaluation; LVD, left ventricular dysfunction; PCI, percutaneous coronary intervention; QoL, quality of life; REVIVED-BCIS2, Revascularization for Ischemic Ventricular Dysfunction_-Study of Efficacy and Safety of Percutaneous Coronary Intervention to Improve Survival in Heart Failure; STICH, Surgical Treatment of Ischemic Heart Failure; STS, Society of Thoracic Surgeons.

Building on the rationale for revascularization, clinical studies have sought to determine the optimal strategy for patients with ischemic LVD. CABG has long been the standard approach, while PCI has emerged as an alternative—particularly for high-risk patients. Landmark trials such as STICH and REVIVED-BCIS2 have significantly influenced our understanding of these strategies (Figure 4).

### 3.1. Evidence for CABG in Left Ventricular Dysfunction

#### 3.1.1. Historical Perspective

Due to the high prevalence of severe CAD in ICM, CABG has traditionally been considered the preferred revascularization strategy. Early evidence supporting CABG was derived from older datasets and subanalyses involving patients with mild LVD. Despite these limitations, surgical revascularization continued to be recommended by guidelines based on the prevailing belief that CABG could improve long-term outcomes in this population [37]. The most robust evidence, however, comes from the STICH trial, the largest randomized controlled trial designed to address whether adding CABG to OMT could reduce mortality compared with OMT alone in patients with CAD and an LVEF below 35% [22,40]. In STICH, patients with significant CAD suitable for surgery were randomized in a 1:1 fashion to receive either OMT alone or OMT plus CABG. The medical regimen included standard therapies such as aspirin, ACE inhibitors or angiotensin II receptor blockers, β-blockers, and statins, while key secondary outcomes included hospitalizations for cardiovascular causes and cardiovascular death. Main exclusion criteria were recent MI considered as responsible for the LVF, important left main disease, and angina classified by the Canadian Cardiovascular Society (CCS) in class III or IV [41].

Although the intention-to-treat analysis at a median follow-up of 56 months did not demonstrate a statistically significant reduction in all-cause mortality, the event curves began to diverge after approximately two years, hinting at a long-term survival benefit with CABG [33]. Nevertheless, this group of patients showed clear evidence of early harm, with an increased 30-day mortality rate (HR 3.19, 95% CI: 1.35–7.52; *p* = 0.008). Furthermore, the early phase of the trial revealed significant risks; the CABG group experienced a notably higher 30-day mortality rate (HR 3.19, 95% CI: 1.35–7.52; *p* = 0.008), with 25% of these patients encountering major complications and nearly 10% either dying or remaining hospitalized 30 days after surgery [42]. These early adverse events underscored the trade-off between the high procedural risk of CABG and its potential long-term benefits.

#### 3.1.2. How Beneficial Is CABG Really?

Subsequent analyses of the STICH data, including per-protocol analyses and the extended follow-up from the STICHES study (median follow-up of 9.8 years), provided further insights. This extended analysis revealed a statistically significant 16% reduction in all-cause mortality in the CABG group compared to the OMT group (*p* = 0.02). In addition, CABG was associated with reductions in secondary endpoints such as cardiovascular death, HF hospitalizations, and overall cardiovascular events [43]. Subgroup analyses within the STICH trial identified that the benefits of CABG were particularly pronounced in patients with three-vessel CAD or those with severely impaired LV function, which were defined, for example, by an LV end-systolic volume index greater than 78 mL/m² or an EF below 27% [44].

Efforts to refine patient selection using myocardial viability imaging were also undertaken. Substudies using SPECT and DSE tried to determine if viability could predict which patients would benefit most from CABG. Despite these efforts, no definitive interaction between viability status and treatment benefit was found after more than 10 years of follow-up [17,30]. One substudy of 399 patients found no significant difference in all-cause mortality between those with or without inducible ischemia, although patients with angina or diabetes appeared to benefit from CABG over a five-year period [45,46,47].

Quality-of-life (QoL) assessments further support the choice of surgical revascularization in ICM. A dedicated STICH QoL substudy, using instruments such as the Seattle Angina Questionnaire and the Kansas City Cardiomyopathy Questionnaire, demonstrated that patients undergoing CABG experienced greater improvements in health-related QoL than those managed with OMT alone [46]. However, limitations such as the retrospective design of these analyses and the lack of prospective trials directly comparing CABG with OMT continue to limit external validation [48].

A prominent hypothesis that has emerged is that the long-term survival benefit of CABG may be attributed not solely to improvements in myocardial contractile function but also to its ability to protect residual viable myocardium from future infarctions [49]. This idea supports the notion that expanding the pool of patients eligible for CABG might further improve clinical outcomes in patients with ICM [50].

### 3.2. Evidence for PCI in Left Ventricular Dysfunction

#### 3.2.1. Prior Evidence Base for PCI

In contrast to CABG, PCI has evolved as a less invasive revascularization strategy, initially applied in patients with less complex CAD and later extended to more challenging scenarios such as multivessel and left main disease. Early experiences with PCI largely involved patients without HF, thereby limiting direct comparisons with CABG in the setting of ICM [51]. Furthermore, many of the randomized trials comparing PCI and CABG in chronic coronary syndromes excluded patients with severe LVD. As a result, significant gaps remain in the understanding of the long-term outcomes of PCI, particularly regarding mortality, hospitalization, and QoL in patients with ICM. Additionally, robust data comparing periprocedural complications, including stent versus graft failure rates and bleeding events (especially in subgroups such as patients with diabetes and renal failure), are still lacking [52,53].

#### 3.2.2. The REVIVED-BCIS2 Trial

To address these uncertainties, the REVIVED-BCIS2 trial was designed to evaluate whether PCI combined with OMT could avoid the early hazards associated with CABG while maintaining the long-term benefits demonstrated in the STICH trial [3,51]. REVIVED is the only randomized trial to date that has questioned the benefit of PCI over OMT in patients with ICM [34]. The trial specifically enrolled patients with extensive and severe CAD, defined by a BCIS (British Cardiovascular Intervention Society) Jeopardy Score of ≥6, and severely impaired LV function (EF ≤ 35%). In addition, eligible patients were required to have at least four dysfunctional myocardial segments at rest with clear evidence of viability, suggesting that revascularization might reverse myocardial hibernation. The recruited population had a median BCIS Jeopardy Score of 10 and a mean LVEF of 27%, characteristics that closely resembled those of the STICH trial population, although with a higher mean age of approximately 70 years. Protocol adherence was excellent, with 96% of patients assigned to PCI undergoing the procedure, and about 10% of patients in the OMT group eventually receiving unplanned revascularization, primarily due to acute MI. Key exclusion criteria included recent MI (within four weeks), decompensated HF, and malignant ventricular arrhythmias within 72 h prior to randomization, ensuring a relatively stable study population.

**Figure 5:**
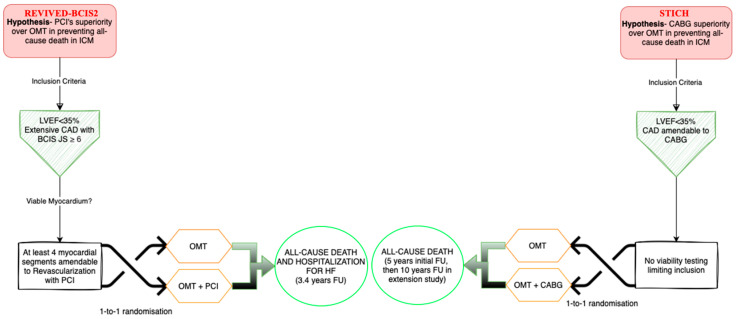
Head-to-head comparison of the REVIVED-BCIS2 and STICH trials protocols. BCIS JS, British Cardiovascular Intervention Society jeopardy score; CABG, coronary artery bypass grafting; CAD, coronary artery disease; FU, follow-up; HF, heart failure; ICM, ischemic cardiomyopathy; LVEF, left ventricular ejection fraction; OMT, optimal medical therapy; PCI, percutaneous coronary intervention; REVIVED-BCIS2, Study of Efficacy and Safety of Percutaneous Coronary Intervention to Improve Survival in Heart Failure; STICH, Surgical Treatment of Ischemic Heart Failure.

Patients were randomized in a 1:1 fashion to receive either PCI plus OMT or conservative management with OMT alone (Figure 5). The primary composite endpoint was death from any cause or hospitalization for HF, while key secondary endpoints included changes in LVEF at 6 and 12 months and QoL scores. At a median follow-up of 41 months, the addition of PCI to OMT did not significantly reduce the primary endpoint compared with OMT alone (37% vs. 38%; HR 0.99; 95% CI [0.78–1.27]; *p* = 0.96). Nevertheless, QoL measures improved significantly in the PCI group at 6 and 12 months, although these differences diminished by 24 months, when outcomes nearly converged with those of the conservative group [3].

#### 3.2.3. Strengths and Limitations of PCI and of the REVIVED-BCIS2 Trial

Beyond the primary composite endpoint, the REVIVED-BCIS2 trial revealed that PCI was associated with a reduction in spontaneous MI, a finding that is consistent with previous studies in patients with stable CAD and is in line with some observations from the STICH trial. However, this reduction did not translate into significant improvements in cardiovascular or all-cause mortality in the short term, similar to the results seen in other revascularization trials in stable ICM [43,54]. The broad inclusion criteria of REVIVED-BCIS2 represent both a strength and a limitation. On one hand, the trial’s design allowed for a more generalizable assessment of PCI in a diverse population with ischemic LVD. On the other hand, many patients in the REVIVED cohort had only intermediate-complexity CAD, which may have limited the trial’s ability to demonstrate a clinically relevant improvement in LV function or a reduction in HF events. Furthermore, the open-label design introduces the possibility that patient-reported outcomes, particularly QoL measures, could be influenced by placebo effects. However, the convergence of QoL scores at 24 months, primarily due to improvements in the OMT group, suggests that these differences were not solely attributable to a transient placebo effect [12,55].

To ensure equitable comparisons, REVIVED-BCIS2 excluded patients with a clear indication for revascularization, thus minimizing the enrollment of individuals with severe angina. In fact, two-thirds of trial participants reported no angina at baseline, which may indicate that HF-related functional impairment masked ischemic symptoms. These methodological factors are critical for interpreting the trial’s outcomes. Moreover, the absence of a CABG arm in REVIVED, along with advancements in contemporary HF treatments such as angiotensin receptor-neprilysin inhibitors (ARNI) and SGLT2 (sodium–glucose cotransporter 2)-inhibitors, underscores the need for updated studies [56,57]. Finally, while CABG with concomitant mitral valve repair is generally preferred in patients with significant functional MR and ICM, PCI may offer an alternative in high-risk patients, with percutaneous edge-to-edge repair reserved for persistent MR [58,59,60,61].

## 4. Comparative Outcomes: PCI Versus CABG

Head-to-head comparisons of PCI and CABG in patients with ICM have shown ambiguous findings, leaving the optimal revascularization strategy uncertain. Most randomized controlled trials comparing the two modalities have been limited to patients with relatively preserved LV function and have often excluded those with complex clinical or anatomical conditions or those who decline surgery, potentially omitting patients who might benefit from either approach [62,63]. A comprehensive revascularization strategy in patients with ICM not only depends on the anatomical extent of CAD but also on the functional significance of individual lesions. The SYNTAX score is a widely used tool that quantitatively assesses the complexity of coronary lesions based on their location, morphology, and distribution. Higher SYNTAX scores typically indicate more complex and diffuse disease, often suggesting that CABG may be a more effective revascularization strategy than PCI [64].

Observational data suggest that survival rates for PCI and CABG are comparable in patients with ICM, though these findings are often derived from studies conducted in patients without severe LVD [65]. A meta-analysis incorporating 18 studies (with a total of 11,686 patients) found that while short-term (30-day) mortality was similar between the two approaches, long-term mortality was lower with CABG in patients with severely reduced EF. Although CABG was associated with a higher early stroke risk, this difference disappeared after 12 months [66].

Longer-term studies have demonstrated the superiority of CABG over PCI in patients with ICM and multivessel CAD. Registries such as CREDO-Kyoto and APPROACH and analyses from Ontario have consistently demonstrated a survival advantage for CABG [8,67,68]. Furthermore, data from STICH and the Swedish Coronary Angiography and Angioplasty Registry (SCAAR) support the preferential use of CABG, particularly in patients with three-vessel disease [65]. Even though CABG carries a higher peri-procedural risk, recent data from Australian and New Zealand registries reaffirm its long-term benefit [69]. This improved results with CABG from randomized controlled trials compared with observational registries could be explained by the fact that the latter ones inherently suffer from selection bias, as patients chosen for PCI may have been deemed poor surgical candidates due to frailty or severe comorbidities. Furthermore, registries often include patients with incomplete revascularization, whereas RCTs ensure more standardized and complete revascularization, favoring CABG outcomes. Additionally, registries may reflect real-world practice, where operator experience and hospital-specific factors influence outcomes, while RCTs eliminate these variables through strict protocol adherence [9,43,70].

On the other hand, observational studies have thus shown that PCI is feasible in patients with severe LV dysfunction, with acceptable in-hospital and long-term mortality rates. PCI’s advantages include symptomatic relief and improved QoL, though its use must be balanced against potential procedural risks such as vascular complications, bleeding, and renal impairment associated with ischemia-related hemodynamic instability [71,72]. In parallel, the principles of functional revascularization have gained prominence recently, particularly with the advent of FFR-guided PCI. This has been shown to improve patient outcomes by ensuring that only lesions that are truly ischemia-inducing are targeted for intervention. This strategy not only minimizes unnecessary stenting but also optimizes resource utilization and clinical outcomes [73,74], proving non-inferior to CABG regarding mortality, stroke, and MI or repeat coronary revascularization after 1 year [75]. In the absence of randomized trials comparing PCI and CABG directly in high-risk patients, registry data suggest that PCI may serve as an acceptable alternative for patients with multivessel disease (MVD) and impaired LVEF who are not candidates for surgical intervention.

The long-term success of revascularization in patients with ICM is closely linked to the extent of myocardial revascularization achieved. A substantial body of evidence indicates that complete revascularization, defined as the successful restoration of blood flow to all significantly stenosed coronary vessels, correlates with a marked reduction in residual ischemia, improved LV function, and a lower incidence of MACE. In contrast, incomplete revascularization (as evidenced by a high residual SYNTAX score) has been associated with ongoing ischemia, adverse ventricular remodeling, and ultimately, higher long-term mortality. It is also recognized that achieving functional completeness of revascularization may be even more important than merely restoring anatomic patency [12].

Outcomes of PCI and CABG are not only determined by the completeness of revascularization; technological advancements in PCI have also played a crucial role in narrowing the gap between the two modalities. In recent years, modern PCI has increasingly incorporated advanced intracoronary imaging techniques, such as intravascular ultrasound (IVUS) and optical coherence tomography (OCT), which facilitate precise lesion assessment, optimal stent sizing, and proper deployment. These imaging modalities enhance procedural accuracy, improve stent apposition, and reduce the risk of restenosis and other complications [76]. Furthermore, in high-risk patient populations, particularly those with severely reduced LV function, the adoption of hemodynamic support devices such as Impella and extracorporeal membrane oxygenation (ECMO) has become integral. These devices provide critical circulatory support during complex interventions, thereby stabilizing patients throughout the procedure and mitigating periprocedural hemodynamic instability. Such support is especially valuable in cases requiring extensive revascularization, allowing for a safer PCI approach even in patients who might traditionally have been managed with CABG [77].

By being a less invasive approach, PCI may as well offer other additional advantages, including lower stroke rates compared with CABG, an observation noted in randomized trials involving patients without HF and in large observational studies of patients with ICM. Furthermore, HF patients, who often have cognitive impairments and multiple cardiovascular risk factors, may be more vulnerable to the neurological sequelae of surgery and cardiopulmonary bypass [78].

Hybrid coronary revascularization (HCR) has emerged as a potential strategy to optimize outcomes in selected patients with multivessel CAD and ICM. HCR combines the durability of CABG, particularly the left internal mammary artery graft on the LAD, with PCI for non-LAD lesions. This approach seeks to strengthen the survival benefit of arterial grafting while minimizing the morbidity associated with full sternotomy and prolonged cardiopulmonary bypass [79].

Recent studies suggest that HCR may be a viable alternative to conventional CABG in high-risk patients, achieving similar long-term outcomes compared to traditional CABG while reducing short-term morbidity, length of hospital stay, and the need for blood transfusions [80]. Additionally, staged HCR, where CABG is performed first, followed by PCI within 60 days, may improve outcomes by allowing optimal recovery before completing revascularization. However, despite these advantages, HCR remains underutilized due to its technical complexity, the need for hybrid-capable surgical teams, and limited RCT data supporting its use over standard CABG or PCI [81].

The optimal revascularization strategy should thus be tailored to the individual patient’s profile. Patients with low to intermediate surgical risk, complex MVD, and preserved or moderately reduced LV function tend to benefit more from CABG. These patients, often characterized by extensive coronary artery involvement (as reflected by higher SYNTAX scores) and significant comorbidities that still permit surgical intervention, may achieve superior long-term survival and improved QoL with CABG. On the other hand, PCI may be preferable for patients deemed at high surgical risk due to advanced age, severe comorbidities, or frailty, particularly when the coronary anatomy is less complex (nevertheless considering the advancements and possibilities of modern high-risk PCI) or when complete revascularization can be achieved percutaneously. In such cases, PCI can offer symptom relief and improved QoL without the higher early procedural risks associated with surgery. Thus, a comprehensive assessment integrating anatomical complexity, comorbidity profiles, and overall surgical risk is essential to determine the most appropriate revascularization modality for each patient [55,68].

### 4.1. But What Do the Guidelines Say?

The heterogeneity and nonuniformity of recommendations for coronary revascularization in ICM reflect the ongoing debate over the optimal strategy. The ESC and the American Heart Association/American College of Cardiology (AHA/ACC) guidelines differ, particularly regarding patients deemed unsuitable for surgery or those who prefer less invasive options. According to the 2018 ESC guidelines, CABG is the main indication for revascularization in patients with significant CAD and low EF, with its benefits supported by up to 10 years of observation from the STICH trial [10]. However, the 2021 ESC guidelines for the diagnosis and treatment of HF downgraded the 2016 recommendation for revascularization in patients with persistent angina despite OMT from Class I to Class IIa and recommended coronary revascularization for heart failure with reduced ejection fraction (HFrEF) in chronic coronary syndromes, with PCI as an alternative to CABG, only as a Class IIb recommendation. The final decision is left to the Heart Team’s discretion, based on a thorough evaluation of the patient’s coronary status, associated comorbidities, and personal preferences [11].

In contrast, the 2021 ACC/AHA/SCAI guidelines continue to recommend CABG as the preferred revascularization strategy for patients with severe LV dysfunction (EF < 35%) and multivessel or complex coronary disease, including left main involvement, assigning a Class 1A recommendation to CABG. Conversely, these guidelines do not provide clear indications for PCI as an alternative to surgery in this context [82].

### 4.2. Current Challenges and Unresolved Questions

Despite the robust data from trials like STICH and REVIVED-BCIS2, guideline recommendations, and advancements in imaging and revascularization techniques, managing ICM remains challenging. A major issue is the diagnostic evaluation of HFrEF, as establishing an ischemic etiology is often underrecognized. Although cardiac MRI with LGE offers valuable prognostic insights and aids in differentiating ischemic from non-ischemic cardiomyopathy, its routine use is limited by availability and cost constraints [48,83].

Decision-making regarding revascularization is another unresolved area. For patients considered ineligible for CABG, often due to advanced age, significant comorbidities, or unfavorable coronary anatomy, the decision typically narrows to PCI versus OMT. Current evidence indicates that prognostic benefits are most evident in carefully selected patients undergoing CABG, whereas PCI is primarily reserved for alleviating refractory angina. Notably, trials such as REVIVED suggest that although PCI does not significantly reduce mortality compared to OMT, it can offer QoL improvements in selected high-risk individuals [46]. The increasing number of high-risk procedures in elderly patients with complex disease and reduced LVEF further complicates the picture. The prognostic impact of high-risk PCI and the optimal use of MCS devices remain uncertain, and current ESC guidelines do not provide specific recommendations for MCS in ICM patients [12]. In this context, the role and indications of high-risk PCI should also be considered, especially since procedural complications, such as vascular complications and in-hospital bleeding, often attributed to the necessity of delivering MCS through large-bore vascular access, contribute to post-procedural morbidity [84].

In recent years, the therapeutic landscape for HF has been revolutionized by the introduction of novel pharmacological agents, particularly SGLT2 inhibitors and ARNI. Landmark trials such as DAPA-HF [57] and EMPEROR-Reduced [85] have demonstrated that SGLT2 inhibitors significantly reduce CV mortality and hospitalization rates in patients with HFrEF. Similarly, the PARADIGM-HF trial established that ARNI not only improves survival but also reduces HF hospitalizations compared to traditional ACE inhibitors [86].

These advancements have redefined OMT and, in many cases, provide robust clinical benefits that may lessen the advantage of invasive revascularization. For instance, patients with ICM who are well-managed with these modern agents might experience significant improvements in symptoms and outcomes, even without revascularization. Consequently, when considering revascularization strategies, it is crucial to evaluate the residual ischemic burden and myocardial viability in the context of contemporary HF therapy. Patients with significant residual ischemia and viable myocardium who continue to experience symptoms despite being on OMT may still derive benefit from CABG or PCI. In contrast, those with minimal residual ischemia might be managed effectively with OMT alone [62,87,88]. Incorporating these modern treatments into risk stratification models allows for a more individualized approach, ensuring that invasive procedures are reserved for patients who are most likely to gain an additional benefit over the impressive outcomes already achieved with state-of-the-art medical therapy [87].

**Figure 6:**
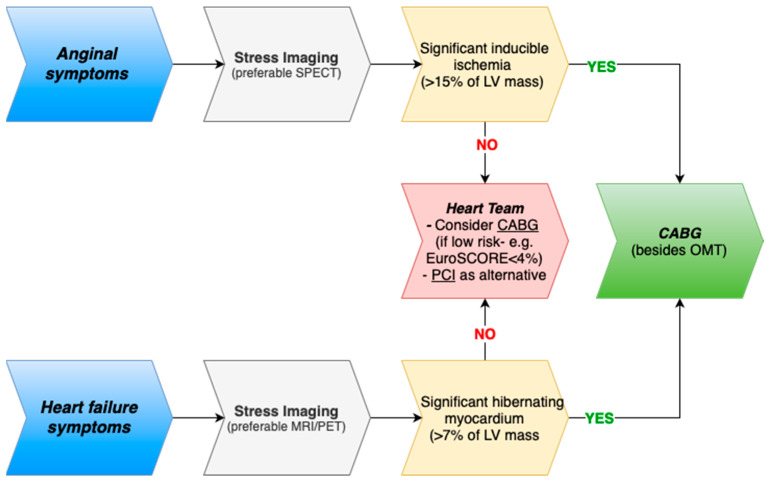
Proposed stepwise management algorithm for ICM patients. CABG, coronary artery bypass grafting; LV, left ventricle; OMT, optimal medical therapy; MRI, magnetic resonance imaging; PCI, percutaneous coronary intervention; PET, positron emission tomography; SPECT, single-photon computed tomography.

Translating these multifaceted issues into clinical practice is challenging, while a stepwise approach may be beneficial (Figure 6). Patients presenting with angina should undergo stress imaging (e.g., DSE, SPECT, PET, or cardiac MRI) to quantify inducible ischemia—where severe ischemia (>15% of LV mass) would favor revascularization with CABG. Conversely, in patients with predominant HF symptoms, evaluation of myocardial viability using PET or MRI is recommended, with revascularization considered when viable myocardium exceeds roughly 7% of LV mass. Finally, procedural risk assessment using tools such as the STS score or EuroSCORE-2 is crucial; a 30-day mortality risk below 4% tends to favor CABG, whereas higher risk or lower anatomical complexity may support PCI after multidisciplinary discussion [61,89].

## 5. Future Directions

Future research in ICM must address these critical uncertainties regarding revascularization. As the landscape of revascularization strategies continues to evolve, several ongoing trials aim to address the limitations of earlier ones by integrating contemporary technologies and revascularization modalities, refined patient selection criteria, and modern therapeutic approaches. Notably, the STICH 3C trial is currently underway and seeks to build on the findings of the original STICH trial by aiming to compare and conclude about the safety and efficacy of state-of-the-art PCI versus CABG in ICM patients with severe MVD, considering improvements and development of modern PCI [90]. Concurrently, studies such as MASS-VI are investigating the comparative effectiveness of surgical revascularization versus OMT in patients with severe ischemic LVD. Together, these initiatives represent a new generation of clinical trials that aim to tailor revascularization approaches more precisely to individual patient profiles, thereby advancing a more personalized and evidence-based framework for managing ICM [48,91].

In parallel with advancements in revascularization techniques, there is growing interest in the application of machine learning (ML) and artificial intelligence (AI) to refine risk stratification and guide treatment decisions in ICM [92]. Recent studies have demonstrated that ML algorithms can process large datasets, including clinical variables, imaging parameters, biomarker levels, and genetic information, to predict outcomes more accurately than traditional risk models. For example, deep learning approaches applied to cMRI imaging have improved the identification of patients at high risk for MACE, while ensemble ML methods have been used to integrate multi-dimensional data to optimize patient selection for revascularization procedures [93,94].

These emerging technologies have the potential to uncover subtle patterns and interactions within complex datasets that are not apparent with conventional statistical methods. Although clinical integration remains in its early stages, preliminary findings suggest that AI-driven models can provide incremental prognostic value and may lead to more personalized therapeutic approaches. As these tools continue to evolve, their incorporation into routine clinical practice could revolutionize the management of ICM by refining risk stratification, enhancing decision-making processes, and ultimately optimizing treatment outcomes [93].

## 6. Conclusions

Outcomes in ICM remain poor, with CABG improving long-term survival in carefully selected patients, while PCI has not shown mortality benefits over OMT. High-risk PCI in patients unsuitable for surgery is increasing, often requiring complex strategies and circulatory support. A multidisciplinary Heart Team discussion is essential for comprehensive management, including viability assessment, complete revascularization, and guideline-directed therapy. Future randomized trials and predictive risk scores are needed to refine strategies and optimize individualized patient care.

## Figures and Tables

**Table 1:** Characteristics of randomized trials for coronary revascularization in ICM. ACS, acute coronary syndrome; BCIS JS, British Cardiovascular Intervention Society jeopardy score; CABG, coronary artery bypass grafting; CAD, coronary artery disease; CCS, Canadian Cardiovascular Society; CI, confidence interval; cMRI, cardiac magnetic resonance imaging; CV, cardiovascular; DSE, dobutamine stress echocardiography; FDG, F-18-fluorodeoxyglucose; F/U, follow-up; HEART, The Heart Failure Revascularization Trial; HF, heart failure; HR, hazard radio LM, left main; LV, left ventricular; LVEF, left ventricular ejection fraction; MI, myocardial infarction; NT-ProBNP, N-terminal pro b-type natriuretic peptide; NYHA, New York Heart Association; OMT, optimal medical therapy; PARR-2, Positron Emission Tomography and Recovery Following Revascularization; PCI, percutaneous coronary intervention; PET, positron emission tomography; QoL, quality of life; REVIVED-BCIS2, Revascularization for Ischemic Ventricular Dysfunction_-Study of Efficacy and Safety of Percutaneous Coronary Intervention to Improve Survival in Heart Failure; SPECT, single-photon computed tomography; STICH, Surgical Treatment of Ischemic Heart Failure.

Trial Acronym	STICH	REVIVED-BCIS2	HEART	PARR-2
Participants	1212	700	138	430
Primary outcome	All-cause death	All-cause death and hospitalization for HF	All-cause mortality	Cardiovascular death, MI, rehospitalization due to cardiac cause within 1 year
Secondary outcome	Cardiovascular death or death by any cause, hospitalization for cardiovascular cause	LVEF at 6 and 12 months, QoL scores, NYHA and CCS class, cardiovascular death, major bleeding, and NT-proBNP levels	Terminated early due to slow recruitment	Time to primary outcome and cardiovascular death
Inclusion criteria	LVEF ≤ 35%, CAD amendable for CABG	LVEF ≤ 35%, extensive coronary artery disease with [BCIS JS Score ≥ 6], viability in at least 4 dysfunctional myocardial segments amenable for PCI	LVEF ≤ 35%, HF ≥ 6 weeks, receiving diuretics, CAD or history of MI, ≥5 viable segments with reduced contractility [assessed by any method]	LVEF ≤ 35%, high suspicion of CAD [from coronary angiogram, previous MI, revascularization, or perfusion imaging]
Exclusion criteria	Recent MI responsible for LV dysfunction, cardiogenic shock within 72 h of randomization, history of CABG, important LM disease, life expectancy < 3 years for noncardiac causes	Acute MI within 4 weeks before randomization, decompensated HF, or malignant ventricular arrhythmias within 72 h prior to randomization	Recent ACS, stroke, valve surgery, angina requiring revascularization, malignant ventricular arrhythmias	Predetermined revascularization or transplantation strategy already planned, prior FDG viability imaging, recent MI [<4 weeks], severe comorbidities, or severe valvular disease requiring surgery
Median F/U	Initial: 4.7 years Extension study: 9.8 years	41 months	59 months	5 years
Imaging technique for myocardial viability	DSE, SPECT	DSE, cMRI, SPECT/PET	DSE, PET, SPECT	PET
Revascularization technique	CABG+OMT [n = 610] vs. OMT [n = 602]	PCI+OMT [n = 347] vs. OMT [n = 353]	CABG [n = 30] and PCI [n = 15] vs. conservative [n = 69]	PET-guided revascularization_CABG [n = 71] and PCI [n = 33] vs. standard care
Outcomes	Initial study: no difference and benefit in all-cause mortality Extension study: Reduction in all-cause mortality [HR 0.84], 95% CI [0.73 to 0.97] and in combined CV hospitalization and death [HR 0.72], 95% CI [0.64 to 0.82] in the CABG group	No significant benefit of revascularization over OMT; improved QoL scores at 6 and 12 months diminished after 24 months when groups outcomes converged	No significant benefit of revascularization regarding mortality and QoL over OMT	No significant difference in composite primary endpoint [cardiac death/ MI/CV hospitalization] between PET-guided and standard strategies at 1- and 5-year follow-up
Limitations	Exclusion of LM disease in the OMT group, crossover between groups, deaths assessed as ‘unknown’	Open-label design bias, largely pauci-symptomatic patients with limited applicability to patients with significant angina or ACS	Small enrollment, underpowered due to early termination	Low protocol adherence that biased the primary outcome analysis

## Data Availability

No new data were created or analyzed in this study.

## Abbreviations

The following abbreviations are used in this manuscript:

ACE	Angiotensin-Converting Enzyme [inhibitors]
ACS	Acute Coronary Syndrome
ACC	American College of Cardiology
AHA	American Heart Association
AI	Artificial Intelligence
ARB	Angiotensin II Receptor Blockers
ARNI	Angiotensin Receptor-Neprilysin Inhibitors
BCIS JS	British Cardiovascular Intervention Society Jeopardy Score
CABG	Coronary Artery Bypass Grafting
CAD	Coronary Artery Disease
CCS	Canadian Cardiovascular Society
cMRI	Cardiac Magnetic Resonance Imaging
CTO	Chronic Total Occlusion
CV	Cardiovascular
DSE	Dobutamine Stress Echocardiography
EF	Ejection Fraction
ESC	European Society of Cardiology
EACTS	European Association for Cardio-Thoracic Surgery
FDG	Fluorodeoxyglucose
FFR	Fractional Flow Reserve
HCR	Hybrid Coronary Revascularization
HF	Heart Failure
HFrEF	Heart Failure with Reduced Ejection Fraction
ICM	Ischemic Cardiomyopathy
IVUS	Intravascular Ultrasound
LAD	Left Anterior Descending Artery
LGE	Late Gadolinium Enhancement
LVD	Left Ventricular Dysfunction
LVEF	Left Ventricular Ejection Fraction
LV	Left Ventricle
MACE	Major Adverse Cardiovascular Events
MCS	Mechanical Circulatory Support
MI	Myocardial Infarction
ML	Machine Learning
MVD	Multivessel Disease
NYHA	New York Heart Association
OCT	Optical Coherence Tomography
OMT	Optimal Medical Therapy
PET	Positron Emission Tomography
PCI	Percutaneous Coronary Intervention
REVIVED-BCIS2	Revascularization for Ischemic Ventricular Dysfunction—Study of Efficacy and Safety of Percutaneous Coronary Intervention to Improve Survival in Heart Failure
SCARR	Swedish Coronary Angiography and Angioplasty Registry
SCAI	Society for Cardiovascular Angiography and Interventions
SPECT	Single Photon Emission Computed Tomography
STICH	Surgical Treatment for Ischemic Heart Failure
STS	Society of Thoracic Surgeons
SGLT2	Sodium–Glucose Cotransporter 2
